# “Colon”ised by the unexpected: a case of extrauterine epithelioid trophoblastic tumour

**DOI:** 10.1186/s13000-025-01617-2

**Published:** 2025-05-24

**Authors:** Shalini Radhakrishnan, Nischitha N. Suvarna, Saraswathy Sreeram, Srirama Bhat

**Affiliations:** 1 Department of Pathology, Kasturba Medical College, Mangalore, Manipal Academy of Higher Education, Manipal, 576 104 Karnataka India; 2 Department of General Surgery, Kasturba Medical College, Mangalore, Manipal Academy of Higher Education, Manipal, 576 104 Karnataka India

**Keywords:** Epithelioid trophoblastic tumour, Gestational trophoblastic disease, Human chorionic gonadotrophin, Colon, Pathology, Immunohistochemistry

## Abstract

**Introduction:**

Extrauterine epithelioid trophoblastic tumour is an exceedingly rare and aggressive form of gestational trophoblastic disease that arises outside the uterus and is characterised by the proliferation of intermediate trophoblastic cells. Unlike more common forms of gestational trophoblastic diseases, such as hydatidiform moles and choriocarcinoma, this entity presents unique diagnostic and therapeutic challenges due to its atypical location and clinical features. Thus far, no documented cases of this entity have been reported in the colon.

**Case presentation:**

We report the case of a 42-year-old woman who presented with complaints of lower abdominal pain and a palpable mass in the left iliac fossa, initially suspected to be an ectopic pregnancy. On radiological evaluation, a provisional diagnosis of gastrointestinal stromal tumour was made, following which the patient underwent a left colectomy with resection and anastomosis, and the excised specimen on comprehensive histopathological and immunohistochemical analysis was diagnosed as a case of extrauterine epithelioid trophoblastic tumour. However, the patient’s condition deteriorated, and she succumbed to the disease one month after the diagnosis.

**Conclusion:**

The rarity of extrauterine trophoblastic tumours contributes to limited clinical experience and treatment protocols, resulting in poor prognoses. This case report highlights the importance of histopathological examination for a confirmatory diagnosis, ensuring timely identification and improving patient outcomes.

## Introduction

With just under 100 cases reported in literature, epithelioid trophoblastic tumours (ETT) represent the least common form of gestational trophoblastic disease (GTD) originating from the chorionic-type intermediate trophoblast. This tumour commonly arises within the lower uterine segment in females of childbearing age, typically between 15 and 48 years. Nonetheless, a noteworthy proportion of cases have been documented among peri- and postmenopausal women with a remote obstetric history [1]. 

In the last few decades, a rising number of studies have focused on cases of ETT occurring in extrauterine sites that are related to ectopic pregnancy, including the fallopian tube, ovary, and broad ligament. Moreover, cases of ETT metastasis may manifest as the primary tumour presentation, with the documented sites involving the lung, liver, gallbladder, kidney, pancreas, spine, vagina, bladder, and ureter [2, 3]. According to the literature, no instances have been reported in the colon. When examined histologically and immunohistochemically, ETT bears a striking resemblance to squamous cell carcinoma (SCC), which could result in misdiagnosis and improper patient treatment. To add further complexity, uncommon somatic carcinomas might exhibit trophoblastic differentiation that can mimic ETT. Therefore, it is imperative to differentiate between ETT and its somatic carcinoma counterparts, given their notable differences in treatment and prognosis. Although clinicopathological factors may suffice in identifying specific histological types in numerous instances, discerning an extrauterine ETT (EETT) from its somatic carcinoma mimickers can pose challenges in the absence of an abnormal gestational history or a uterine mass lesion [4]. 

We report the case of a middle-aged woman who came with complaints of lower abdominal pain and a palpable mass in the left iliac fossa, which was initially thought to be a routine case of ectopic pregnancy but was later diagnosed as EETT arising from the descending colon on comprehensive histopathological and immunohistochemical evaluation. This case report documents the first known instance of EETT arising from the colon, adding to the limited literature on this entity and aiming to increase awareness amongst clinicians and pathologists.

## Clinical presentation

A 42-year-old woman came with complaints of lower abdominal pain for two months, which was insidious in onset, gradually progressive, radiating to the left side and occasionally relieved by taking medication. She was a known diabetic and on medication for 10 years. She also had a previous history of a lower-segment Caesarean section (LSCS) along with a myomectomy for fibroid removal, which was performed a decade ago.

A general physical examination revealed anaemia. On abdominal examination, tenderness was elicited in the left iliac fossa with a palpable mass measuring 5 × 4 cm, which was firm in consistency. The lower border of the mass could not be appreciated on palpation; its mobility was restricted, and abdominal guarding was elicited. A pelvic examination was done, which yielded no significant findings. However, on performing a qualitative urine human chorionic gonadotrophin (hCG) test, the result was determined to be positive. Considering these findings, a clinical diagnosis of an ectopic gestation was made, and further evaluation was advised.

Routine haematological parameters were evaluated, and they showed moderate anaemia. Relevant biochemical parameters were also assessed; serum lactate dehydrogenase (LDH) levels were elevated (370 U/l), while the rest were unremarkable. An abdominal and pelvic ultrasound was performed for confirmation, which, however, showed a solid to cystic heteroechoic lesion in the left lumbar-iliac region measuring 8 × 7.3 × 6 cm with internal septations, solid components, and echogenic debris and minimal vascularity, with no evidence of a gestational sac. A magnetic resonance imaging (MRI) of the abdomen and pelvis with intravenous contrast was done, which showed a heterogeneously enhanced, lobulated, oval, thin-walled lesion measuring 6.9 × 6.5 × 4.8 cm. The lesion was predominantly cystic, with peripheral solid components involving the small bowel at the left lumbar region. Anteriorly, the lesion was appreciated up to the left anterior abdominal wall, which displaced the descending colon medially, abutting the left psoas and iliac muscle postero-laterally. The uterus and adnexal structures appeared unremarkable, and no other lesions were observed on imaging. Based on these findings, a preliminary diagnosis of gastrointestinal stromal tumour (GIST) was given, and pathological correlation was advised. Serum tumour markers were assessed for further evaluation, which showed levels of carcinoembryonic antigen (CEA), CA 125 and CA 19 − 9 to be within normal limits. In contrast, the level of β-hCG was mildly elevated (370 mIU/l).

An explorative laparotomy was performed under general anaesthesia, and intra-operatively, a neoplastic growth was appreciated in the descending colon, which was posterior to the left psoas muscle and adherent to the anterior abdominal wall, not involving the uterus and other adnexal structures. A left colectomy was performed with anastomosis, and the resected segment was submitted for histopathological examination.

**Fig. 1:**
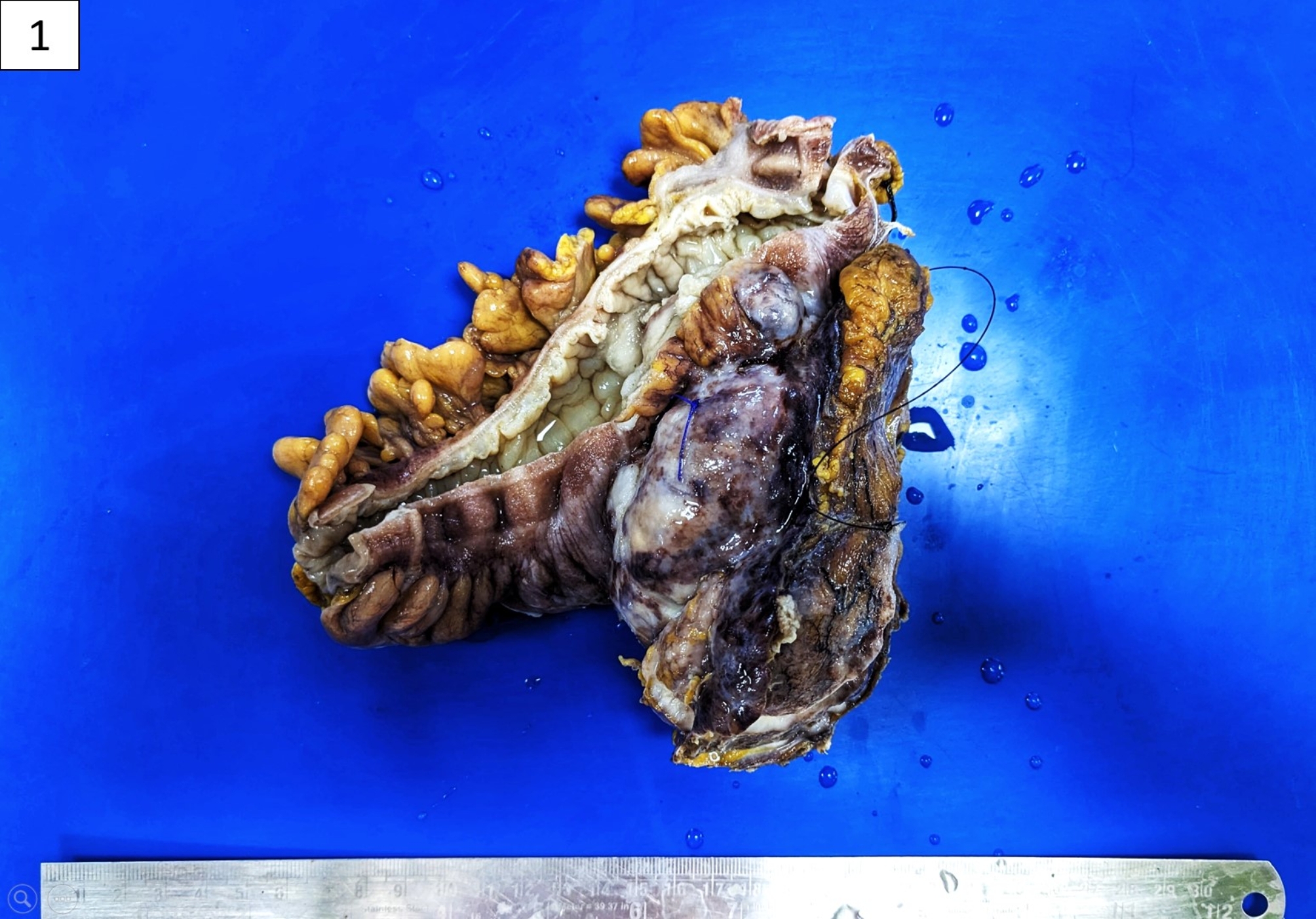
Specimen of left hemicolectomy: haemorrhagic tumour noted on the serosal aspect of descending colon

Grossly, a specimen of left hemicolectomy was received, comprising segments of the descending colon and sigmoid colon measuring 17 cm in length. An exophytic tumour measuring 10 × 7 × 5.5 cm was noted laterally on the serosal surface, located 3 cm from the proximal resected end and 7 cm from the distal mucosal resected end. Upon slicing serially, the specimen revealed 28 lymph nodes and an ulcer measuring 0.5 cm on the mucosal surface. The outer surface of the tumour contained numerous congested blood vessels. Its cut surface was solid to cystic in consistency, and a firm, pale white nodular lesion was observed in the centre, which extended up to the lateral margin. A haemorrhagic cyst was also appreciated at the centre, measuring 6 × 5.5 × 5 cm in diameter (Fig. 1).

**Fig. 2:**
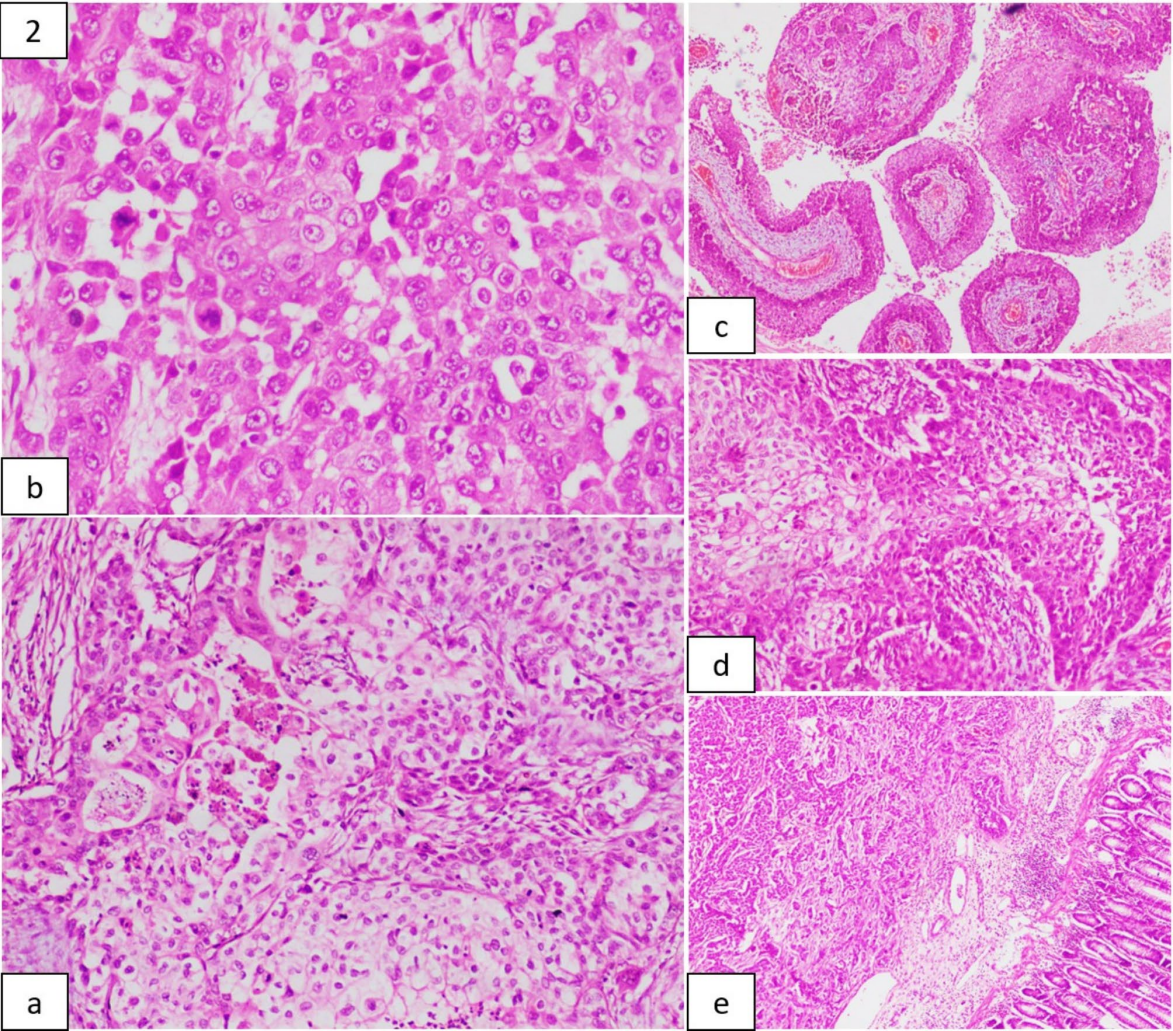
Histopathological Examination of Tumour (from **a**): Composed of epithelioid cells in glandular pattern with foci of necrosis; Tumor cells showing nuclear pleomorphism and numerous mitotic figures; Tumor cells arranged in squamoid configurations; Mucosa uninvolved by the tumour. (H&E stain; 200*x*, 100*x* and 40*x* magnification)

Representative sections from the specimen were submitted for further evaluation, which, on histological examination, showed ulcerated colonic mucosa with a tumour that extensively infiltrated the submucosa, muscularis propria and serosa, which comprised of neoplastic cells arranged in solid sheets, and nests. These cells contained abundant eosinophilic to vacuolated cytoplasm with round to oval nuclei, which exhibited moderate to marked nuclear polymorphism, bearing irregular nuclear contours, and each had a single prominent eosinophilic nucleolus. Certain nests of tumour cells that showed squamoid differentiation were appreciated. Scattered multinucleated tumour giant cells and high mitotic activity (20 to 25 mitoses/10 high-power fields (HPF)) were observed (Fig. 2). Numerous cysts lined by these tumour cells were also appreciated. The surrounding stroma showed a desmoplastic reaction with extensive necrosis and focal myxoid areas. Lymphovascular invasion was present in the specimen. All regional lymph nodes obtained were found to be free of the tumour. Based on these findings, a morphological diagnosis of poorly differentiated malignant neoplasm was given.

**Fig. 3:**
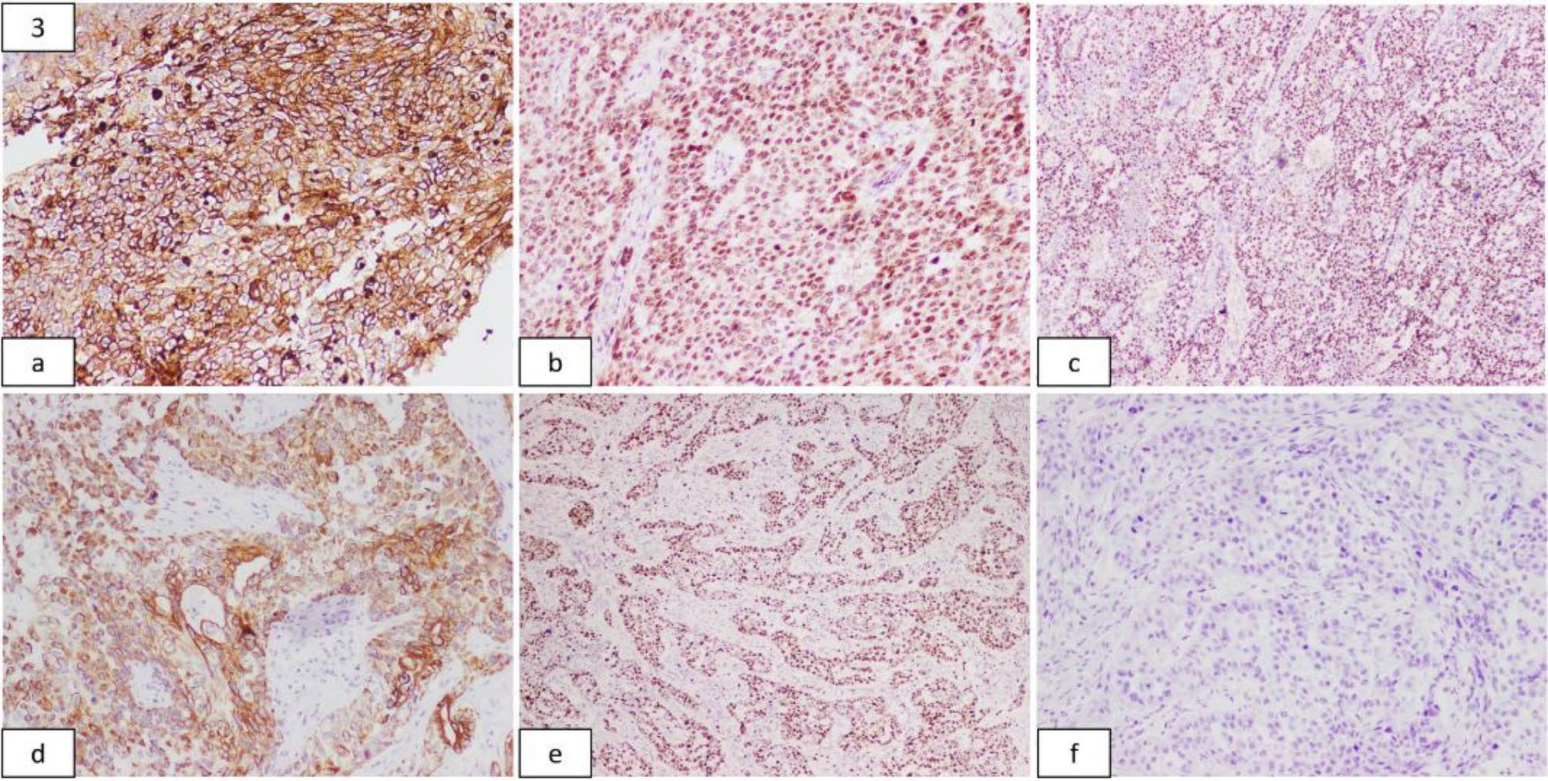
Immunohistochemical Stains (from **a**): Tumour showing strong membranous positivity for CD 10; Positive nuclear staining for GATA3 and p63; Tumor cells showing diffuse cytoplasmic positivity for CK 7; Ki67 proliferation index is 55%; No discernible positivity seen for CK 20. (100*x* and 40*x* magnification)

An immunohistochemical analysis revealed the tumour to be diffusely positive for GATA3, CK 7, p63, and CD 10. Immunostains for cytokeratins 5/6 and 20, p16, thyroid transcription factor-1 (TTF-1), chromogranin, and calretinin showed no reactivity. The MIB labelling index was 55% (Fig. 3). The positive and negative external controls exhibited appropriate reactivity to all employed markers. Considering the findings mentioned above, along with the clinico-radiological details, a final diagnosis of EETT was made.

The postoperative course was uneventful, and upon achieving haemodynamic stability, the patient was discharged and advised to consult a medical oncologist. However, she was lost for follow-up later and, unfortunately, succumbed to the disease one month after the diagnosis.

## Discussion

The symptoms of ETT resemble those of other types of GTD, with a majority of patients (57–67%) presenting with abnormal vaginal bleeding at the time of diagnosis. It is common for patients to display normal to slightly elevated serum β-hCG levels (< 2,500 mIU/mL) at the time of presentation. However, there are documented cases of markedly elevated serum β-hCG levels, particularly in EETT. According to the literature, the clinical features observed in these cases include a previous history of molar pregnancy, coincidental findings on physical examination and radiological imaging, episodes of haemoptysis and cough, back pain and abdominal discomfort [5, 6]. Twenty-seven cases of EETT have been documented, twelve of which highlight the lungs as the primary site [2, 4, 7]. There have been no reported cases of EETT with the colon as the primary site.

A precise mechanism for the pathogenesis of EETT has not been established; clinical cases are the only documented evidence [4]. Upon microscopic examination, this tumour displays a nodular growth pattern comprising a uniform population of mononuclear chorionic-type intermediate trophoblastic cells arranged in cords and nests, accompanied by an eosinophilic, fibrillar, hyaline-like matrix and necrosis. The neoplastic cells usually have a moderate amount of finely granular, eosinophilic to clear cytoplasm, distinguished by distinct cellular membranes and round nuclei with prominent nucleoli and minimal nuclear atypia, with the mitotic activity typically falling between 0 and 9 mitoses/10 HPF, with a mean value of 2 mitoses/10 HPF. Dystrophic calcification and scattered multinucleated giant cells may be appreciated. These cells exhibit diffuse immunoreactivity for markers such as hydroxyl-δ-5-steroid dehydrogenase (HSD3B1), human leukocyte antigen G (HLA-G), p63, cyclin E, α-inhibin, and GATA3. However, conventional trophoblastic markers, such as human placental lactogen (hPL), β-hCG, and melanoma cell adhesion molecule (Mel-CAM or CD146), exhibit focal positivity, and the ki67 proliferation index is usually greater than 10%. Cytokeratins such as CK 7 and CK AE1/AE3 are typically positive. Nonetheless, these cells do not express CK 20 [8, 9]. 

From a clinical perspective, it is crucial to differentiate between ETT, other forms of GTD, and other epithelial malignancies to devise appropriate therapeutic strategies. Mitotically active choriocarcinomas, characterised by the presence of mononuclear trophoblasts encircled by multinucleated syncytiotrophoblasts and placental site trophoblastic tumours (PSTT), distinguished by their distinct weaving pattern of invasion, can be distinguished from ETT based on morphological features. Immunoreactivity to cytokeratins combined with the lack of expression of smooth muscle markers helps distinguish it from smooth muscle tumours with epithelioid morphology. Meanwhile, the eosinophilic matrix could resemble keratin, leading to potential confusion between ETT and an epithelial malignancy like squamous cell carcinoma (SCC). α-inhibin and CK 18 are noteworthy for their expression in ETT but not in SCC, potentially aiding the distinction between them. Moreover, unlike ETT, where the Ki-67 labelling index is generally low, ranging from 10 to 25%, SCC consistently demonstrates a markedly elevated Ki-67 labelling index, exceeding 50%. In contemplating the differential diagnosis associated with potential germ cell neoplasms, one must consider that ETT can express specific cytokeratins, placental alkaline phosphatase (PLAP) and CD117 (c-kit) [1, 6, 9]. However, in a study conducted by Niu N et al., summarising four puzzling cases of EETT, the histological features bore a striking resemblance to various somatic carcinomas with trophoblastic differentiation. Moreover, immunohistochemically, all four tumours exhibited varying expression of trophoblastic markers like GATA3, hPL, α-inhibin, PLAP, β-hCG, and p63, in addition to cytokeratin expression. Ancillary techniques such as short tandem repeat (STR) genotyping have been advocated in such scenarios [4]. 

Patients diagnosed with ETT tend to metastasise to the lungs, bones, and spine, resulting in an average mortality rate ranging from 10 to 24%. The prognosis may be further compromised by an elevated mitotic rate (> 6 mitoses/10 HPF), age of more than 40 years and a duration exceeding 4 years since the last pregnancy. Multifocal lesions, infiltration of the myometrium, and serosal involvement are the additional unfavourable factors noted in ETT [1, 10]. The behaviour of ETT remains ambiguous due to its recent classification as a unique form of trophoblastic disease and limited access to long-term follow-up data. These tumours are chemo-resistant and are, therefore, treated surgically. The suggested approach involves performing a hysterectomy alongside a pelvic lymph node dissection due to the potential for lymphatic spread and metastasis to the lymph nodes. However, chemotherapy is recommended for patients presenting with metastatic disease and in cases of nonmetastatic disease characterised by unfavourable prognostic indicators. Currently, it is recommended to use a platinum-based regimen, such as EMA-EP or a doublet of paclitaxel/cisplatin-paclitaxel/etoposide.[1, 2, 10] Due to the rarity of EETT and the long-term nature of clinical trials, no standardised and definitive chemotherapy regimens are in place for its treatment, whether metastatic or not. In recent years, there has been a remarkable upsurge of interest in the application of immunotherapy in individuals with trophoblastic disease, primarily attributed to the substantial expression of PD-L1 receptors in trophoblastic cells. Studies have reported on the effective use of pembrolizumab in treating different types of GTD, including choriocarcinoma. Two cases of EETT with extensive metastasis have been reported, and the patients were treated using pembrolizumab, leading to a reduction in disease burden and complete resolution of metastasis [11]. Therefore, the potential treatment modalities may expand, leading to a more favourable outcome in the coming years.

## Conclusion

EETT is a highly uncommon type of GTD known for its erratic biological behaviour. Recognition of this entity should be prompted upon identification of a focal lesion in anatomical locations outside the uterus and concurrent elevation of β-HCG serum levels in patients manifesting with localised masses and possible episodes of irregular vaginal bleeding after previous gestations. Histopathological and immunohistochemical findings ensure a precise and reliable diagnosis. The extraction of the tumour mass continues to be the primary therapeutic approach. Although EETT has been recognised for its relatively limited response to chemotherapy, additional research is imperative to elucidate the precise impact of chemotherapy in managing this condition. Further research is warranted to identify the most suitable chemotherapeutic approach for this disease.

## Data Availability

Data sharing does not apply to this article as no new datasets were generated or analysed during the current study.

## References

[1] Marquina G, Szewczyk G, Goffin F. The rare of the rarest: PSTT, ETT, APSN. Gynecol Obstet Invest. 2024. 10.1159/000536494 38281479

[2] Gallardo J, Hummel K, Siatecka H, McCluskey K, Sunde JS, Elshaikh A, et al. Epithelioid trophoblastic tumor presenting as an Adnexal Mass: report of a diagnostically challenging case. Int J Surg Pathol. 2023;31(5):651–5. 35946122 10.1177/10668969221117983

[3] Chohan MO, Rehman T, Cerilli LA, Devers K, Medina-Flores R, Turner P. Metastatic epithelioid trophoblastic tumor involving the spine. Spine. 2010;35(20):E1072–5. 20802395 10.1097/BRS.0b013e3181d7696b

[4] Niu N, Ordulu Z, Burak Z, Buza N, Hui P. Extrauterine epithelioid trophoblastic tumour and its somatic carcinoma mimics: short tandem repeat genotyping meets the diagnostic challenges. Histopathology. 2024;84(2):325–35. 37743102 10.1111/his.15054

[5] Dash SS, Sakhadeo U, Karmarkar S, Mittal N, Menon S, Rekhi B, et al. Epithelioid trophoblastic tumor: a case series. Indian J Pathol Microbiol. 2023;66(1):148–51. 36656227 10.4103/ijpm.ijpm_212_22

[6] Kumar A, Diwan H, Sharma A, Kamboj M. Epithelioid trophoblastic tumors: a diagnostic challenge. Int J Gynecol Cancer. 2023;33(1):132–4. 36423957 10.1136/ijgc-2022-004027

[7] Okereke IC, Chen S. Primary epithelioid trophoblastic tumor of the lung. Ann Thorac Surg. 2014;97(4):1420–1. 24694417 10.1016/j.athoracsur.2013.07.031

[8] WHO classification of female genital tumours. 5th ed. Vol. 4. *Lyon (France): International Agency for Research on Cancer*; 2020.

[9] Gwin K. Epithelioid Trophoblastic Tumor. In: Gestational Trophoblastic Disease. *New York, NY: Springer New York*; 2012. pp. 105–25.

[10] Liu W, Zhou J, Yang J, Huang X. A Multicenter Retrospective Study of Epithelioid Trophoblastic Tumors to identify the outcomes, prognostic factors, and therapeutic strategies. Front Oncol. 2022;12:907045. 35677151 10.3389/fonc.2022.907045 PMC9169038

[11] Bell SG, Uppal S, Sakala MD, Sciallis AP, Rolston A. An extrauterine extensively metastatic epithelioid trophoblastic tumor responsive to pembrolizumab. Gynecol Oncol Rep. 2021;37:100819. 34258359 10.1016/j.gore.2021.100819 PMC8258853

